# Compositional Analysis of Grape Berries: Mapping the Global Metabolism of Grapes

**DOI:** 10.3390/foods13233716

**Published:** 2024-11-21

**Authors:** Huanteng Hou, Yufei Li, Shen Zhou, Ran Zhang, Yuanyue Wang, Long Lei, Chenkun Yang, Sishu Huang, Hang Xu, Xianqing Liu, Min Gao, Jie Luo

**Affiliations:** 1 National Key Laboratory for Tropical Crop Breeding, School of Breeding and Multiplication (Sanya Institute of Breeding and Multiplication), College of Tropical Agriculture and Forestry, Hainan University, Sanya 572025, China; 2 Yazhouwan National Laboratory, Sanya 572025, China; 3 Hainan Seed Industry Laboratory, Sanya 572025, China; 4 State Key Laboratory for Crop Stress Resistance and High-Efficiency Production, College of Horticulture, Northwest A&F University, Yangling 712100, China; 5 Key Laboratory of Horticultural Plant Biology and Germplasm Innovation in Northwest China, Ministry of Agriculture, Northwest A&F University, Yangling 712100, China

**Keywords:** grapes, French paradox, bioactive compounds, nutrients, metabolic profile, variations

## Abstract

To characterize the nutrients and bioactive compounds in grape berries and to explore the real cause of the “French paradox” phenomenon, we performed metabolomic analysis of 66 grape varieties worldwide using liquid chromatography–tandem mass spectrometry (LC-MS). A nontargeted metabolomics approach detected a total of 4889 metabolite signals. From these, 964 bioactive and nutrient compounds were identified and quantified, including modified flavonoids, medicinal pentacyclic triterpenoids, vitamins, amino acids, lipids, etc. Interestingly, metabolic variations between varieties are not explained by geography or subspecies but can be significantly distinguished by grapes’ color, even after excluding flavonoids and anthocyanins. In our analysis, we found that purple grape varieties had the highest levels of key bioactive components such as flavonoids, pentacyclic triterpenes, and polyphenols, which are thought to have a variety of health benefits such as antioxidant, anti-inflammatory, and antitumor properties, when compared to grapes of other colors. In addition, we found higher levels of vitamins in red and pink grapes, possibly explaining their role in preventing anemia and scurvy and protecting the skin. These findings may be a major factor in the greater health benefits of wines made from purple grapes. Our study provides comprehensive metabolic profiling data of grape berries that may contribute to future research on the French paradox.

## 1. Introduction

Grapes are considered one of the most important economic horticultural crops in the world [1]. They are not only a popular fresh fruit but also serve as raw materials for wine, grape juice, and raisins, driving international trade and economic development. In addition to their unique flavor, grapes offer a variety of health benefits. These include cancer prevention, diabetes prevention, cardiovascular disease prevention, and anti-inflammatory properties [2,3]. The “French paradox” is a remarkable phenomenon that demonstrates the unexpectedly low incidence of cardiovascular disease among the French, despite their consumption of high-fat, high-cholesterol cuisine [4,5]. This phenomenon has garnered considerable attention and interest in the realm of health and nutrition research, particularly due to the widely held belief that the red wine consumed by the French contains beneficial components for cardiovascular health. It also implies that grapes contain highly effective nutrients and bioactive compounds. In recent years, a significant amount of research has focused on exploring the antioxidant activity of compounds in grapes to understand their potential health benefits. Studies have found that polyphenolic compounds such as flavonoids, phenolic acids, and stilbenes present in grapes are considered one of the important reasons for their health-promoting functions [6,7,8].

Grapes are rich in flavonoids, primarily consisting of flavanols, flavonols, and anthocyanins [9]. Flavanols, which are characterized by a hydroxyl group at C-ring position 3, have antioxidant, anti-inflammatory, and cardiovascular benefits [10]. Clinical studies indicate that these compounds support vascular health, enhance blood flow, reduce clotting, and may improve cognitive function [11]. Flavonols are distinguished by a C2–C3 double bond, a carbonyl group at C4, a ringed C ring, and hydroxyl substitution at C3, which differentiates them from other flavonoids. These compounds are commonly found in various fruits and vegetables. In addition to their antioxidant and anti-inflammatory effects, flavonols also demonstrate significant anticancer properties and contribute to enhanced visual function [12,13]. For instance, quercetin has been demonstrated in several studies to inhibit the proliferation of various cancer cells, including those associated with breast, lung, and colon cancers. Additionally, it helps alleviate visual fatigue [14]. Anthocyanins, a type of flavonoid, possess a 2-phenylbenzopyran ring with various substituents and share functional characteristics with other flavonoid metabolites [15]. Their color changes with pH, allowing them to exhibit different hues in varying environments. Red wine, made from purple and red grapes that are rich in anthocyanins, displays a distinctive purplish-red color. The high concentration of anthocyanins endows red wine with stronger antioxidant properties compared to other types, offering significant health benefits.

Stilbenes are non-flavonoid polyphenolic compounds derived from the phenylpropanoid pathway and are classified as diphenylethylene derivatives. Research indicates that resveratrol (RES) has antioxidant and anti-inflammatory properties, which positively influence cardiovascular health in animal models [16]. Consequently, resveratrol has long been regarded as a key factor in the “French paradox”. However, recent studies have uncovered issues such as misleading promotions, data fraud in research regarding its effects, and findings that are primarily limited to animal and cell experiments. Notably, beneficial cardiovascular trial data have only emerged from long-term, high-dose intake in these models [17,18]. As a result, scientists have considered the significant controversy surrounding the role of stilbenes, such as resveratrol, in grapes, as well as the possibility that grapes may also contain other biologically active compounds. They have proposed a hypothesis: there may be undiscovered key biologically active compounds present in grapes [19,20].

In medicinal plants, various bioactive compounds have been identified with significant preventive effects against cancer, diabetes, cardiovascular diseases, inflammation, and aging. These compounds are uniquely distributed across different plants; for instance, potent anticancer compounds (PMFs) are found in *Scutellaria baicalensis*, antidiabetes flavonoid glycosides (MbA) in montbretia (*Crocosmia x crocosmiiflora*), and anticardiovascular components (pentacyclic triterpenoid saponins) in Panax ginseng. They play vital roles in enhancing immunity, supporting nervous system and muscle function, regulating metabolism, combating oxidative stress, and extending lifespan [21,22]. However, limitations in detection technology and research strategies hinder the exploration of similar pharmaceutical active ingredients in grapes and other plants. This restricts our comprehensive utilization and development of these resources.

Therefore, a comprehensive chemical and biological characterization of these compounds is essential. Identifying and describing the chemical structures, properties, and functions of grape compounds not only elucidates their roles in physiological processes but also assists in evaluating their potential health benefits [23,24]. However, the low concentrations of these compounds complicate detection and analysis. Furthermore, the complex metabolic pathways in grapes involve multiple interacting compounds, which further challenge comprehensive exploration and identification. To address these issues, researchers must employ advanced analytical techniques in conjunction with integrated bioinformatics methods.

Nontargeted metabolomics serves as a powerful tool for the comprehensive analysis of metabolites in biological samples. This approach does not rely on any predefined metabolites, thereby facilitating the discovery of new or unknown compounds [25]. In plant research, untargeted metabolomics has unveiled a wide array of bioactive substances with beneficial effects on human health, including, but not limited to, polyphenols, flavonoids, vitamins, and amino acids. However, to date, research on metabolites in grapes has been limited to a few specific classes of metabolites and even fewer bioactive compounds. Furthermore, the assembly of the grape genome provides a valuable resource for functional omics studies. It has also been demonstrated that genome-wide association analyses, in conjunction with metabolomic data, are powerful tools for reconstructing metabolic pathways across different materials. By employing these techniques, researchers can explore the components of grapes that play significant roles in preventing cancer, diabetes, cardiovascular diseases, and inflammation and promoting longevity, thereby enhancing the medicinal value of grapes and elucidating their mechanisms of action and metabolic pathways.

In this study, we performed a comprehensive metabolic profiling of primary and secondary metabolites from grapes and identified/annotated and quantified 964 of these metabolites using an LC-MS-based untargeted metabolomics approach. We potentially identified multiple new metabolites with therapeutic properties for a wide range of diseases, including modified flavonoids and medicinal pentacyclic triterpenoids, which may have potential therapeutic effects against diseases. These compounds were significantly higher in purple grapes, consistent with the phenomenon observed in the “French paradox”.

## 2. Material and Methods

### 2.1. Plant Materials

To investigate grape metabolomics, grape fruits from different cultivars and wild varieties from various grape production areas worldwide were analyzed. A total of 66 grapevine germplasm resources were used in this study, including cultivar species (*Vitis vinifera*) and wild species (*V. ficifolia, V. cinerea*, *V. rupestris*, *Ampelopsis glandulosa,* and *V. labrusca*). These samples were mainly collected from germplasm resource centers in Beijing, Shaanxi, and Shanghai. The grapes were cultivated in the grape germplasm resource garden at Northwest A&F University, which is situated in a continental monsoon semi-humid climate zone. The average annual temperature is 12.9 °C, with annual precipitation ranging from 635.1 to 663.9 mm, an average annual evaporation of 1500 mm, and approximately 2160 h of sunlight per year. The frost-free period lasts for 210 days. The vines from which the grapes were harvested were approximately 5 years old. Vineyard management practices, including irrigation, pest control, and fertilization, adhered to local standards without any special regulations regarding water or fertilizer. In winter, branches were pruned and the garden was cleared to reduce overwintering eggs of insects and prevent diseases. In late March, before grape germination, the entire garden was sprayed with 5% sulfur to prevent overwintering fungi, bacteria, and insect eggs. In mid-to-late April, a solution of 50% fumonisin (2000 times) + 10% bifenthrin (3000 times) was used to control black pox and green blind stinkbugs. Before and after grape blossoming in May, a mixture of 25% pyraclostrobin (2000 times) + 22% imidacloprid (1500 times) was applied to prevent brown blight and gray mold. Also in May, before and after flowering, a combination of 25% pyrazoxystrobin (2000 times), 22% imidacloprid (1500 times), and 70% imidacloprid (7500 times) was used for brown blight and gray mold prevention. From June to July, a solution of 30% benzylpyrazole ester (2000 times), 50% allylmorpholine (3000 times), and 5% amidacloprid (5000 times) was applied to protect against anthracnose, white rot, and mites. In September, steps were taken to prevent mildew using fungicides primarily based on copper preparations or powders. In addition to watering, according to the tree’s growth potential, budding fertilizer, pre-flowering fertilizer, fruit expansion fertilizer, color change fertilizer, post-harvest fertilizer, etc., were applied. In the early stages of growth, a nitrogen-based fertilizer with phosphorus and potassium fertilizer was applied; in the middle of the process, a phosphorus-based fertilizer was applied; and in the latter part of the process, the main application was potash with an appropriate amount of nitrogen-based fertilizer. About 15 kg per mu of fertilizer was applied each time.

The grapes were harvested for analysis when they reached the desired maturity, as indicated in Supplementary Table S1, which was 135 days after flowering. Grape berries from each variety were randomly selected from different vines throughout the vineyard (*n* > 30), with three replicates for each variety. All samples were immediately placed in liquid nitrogen at −80 °C for preservation and subsequently vacuum freeze-dried. Vacuum freeze-drying of grape samples was conducted using a Millrock EPICTM freeze-dryer (Millrock Technology, Kingston, NY, USA, https://www.millrocktech.com/ (accessed on 5 January 2024)). The overall process extended for approximately 7 days. The specific conditions employed during the process were as follows: vacuum level: 300 mTorr at −30 °C for 2920 min; vacuum level: 200 mTorr at −20 °C for 120 min; vacuum level: 100 mTorr at −20 °C for 2100 min; vacuum level: 50 mTorr at −10 °C for 120 min; vacuum level: 30 mTorr at −10 °C for 2400 min; vacuum level: 30 mTorr at 0 °C for 720 min; vacuum level: 30 mTorr at 25 °C for 120 min; and vacuum level: 30 mTorr at 26 °C for 1400 min.

### 2.2. Chemical Reagents

Chromatographic-grade acetic acid and methanol were purchased from Merck (Darmstadt, Germany). The Milli-Q water was purified using a Millipore purification system (Millipore Corporation, Burlington, MA, USA). The internal standard used in this study was lidocaine, which was bought from Shanghai New Asiatic Pharmaceuticals Co., Ltd. (Shanghai, China) (http://www.xinyapharm.com/). Oleanolic acid and quercetin 3-o-β-(6″-O-E-P-coumaryl glucoside)-7-O-β-glucoside were prepared as external standards by Sigma-Aldrich, in the United States (http://www.sigmaaldrich.com/united-states.html, accessed on 5 January 2024). All standards were dissolved in methanol to make stock solutions, diluted to appropriate concentrations with 70% methanol aqueous solution (methanol:H_2_O, 7:3, *v*/*v*), and detected using LC-MS. All standard stock solutions were stored at −20 °C in the dark.

### 2.3. Metabolite Sample Preparation

Fresh fruit were collected in 50 mL centrifuge tubes and quickly frozen in liquid nitrogen and freeze-dried. Three biological replicates were collected for each species. The samples were ground into powder using a grinder machine (MM400, Retsch, Haan, Germany) with steel balls at 28 Hz for 56 s or more. Then, 0.05–0.1 g of sample powder was suspended with 70% methanol water solution in the ratio of 1:10,000. Next, the samples were extracted by ultrasonic wave for 10 min at 50 Hz three times [26]. At the end of each time, vortex vibration and mixing were required.

### 2.4. Metabolomic Detection

Nontargeted metabolic profiling analyses were performed with Q Exactive Focus Orbitrap LC-MS/MS (Thermo Scientific, Waltham, MA, USA). The scanning mass ranged from *m*/*z* 100 to 1000 with an accumulation time of 0.10 s. The scanning mode was full MS/ddMS2. The recorded data were processed with compound discoverer (CD) 3.3 software to obtain the mass-to-charge ratio, retention time, and the MS/MS2 information of all detected compounds. Following the approach pioneered in plants by Morreel et al. [27], these features were used to screen the data in the literature and database. The annotated metabolites were further identified with the help of available standards. The multiple reaction monitoring (MRM) mode with QTRAP 6500+ LC-MS/MS (Shimadzu, Kyoto, Japan) was used for targeted metabolome analyses. The detection window was set to 80 s, and the targeted scanning time was 1.5 s. The original data were processed by Multi Quant 3.0.3 software. The chromatographic column was a C18 column (Shim-pack GLSS C18, 1.9UM, 2.1 × 100, Shimadzu). Mobile phase A was 0.04% acetic acid–water solution, and mobile phase B was 0.04% acetic acid–methanol solution. The flow rate was 0.4 mL/min. The qualitative and quantitative chromatographic conditions were consistent.

### 2.5. Statistical Analysis

The relative signal strength of the metabolites was divided and normalized according to the internal standard (0.1 mg L^−1^ lidocaine). Z score normalization was applied to normalize the expression values of metabolites, followed by hierarchical clustering analysis and PCA. Differentially accumulated metabolites (DAMs) were screened using Student’s *t*-test. Metabolites with *p* < 0.05 were considered DAMs. The differences between the metabolites in 66 grapes were calculated using nested ANOVA in the R 4.4.0 package.

## 3. Results

### 3.1. Nontargeted Metabolic Signal Acquisition of Grape Samples

**Figure 1:**
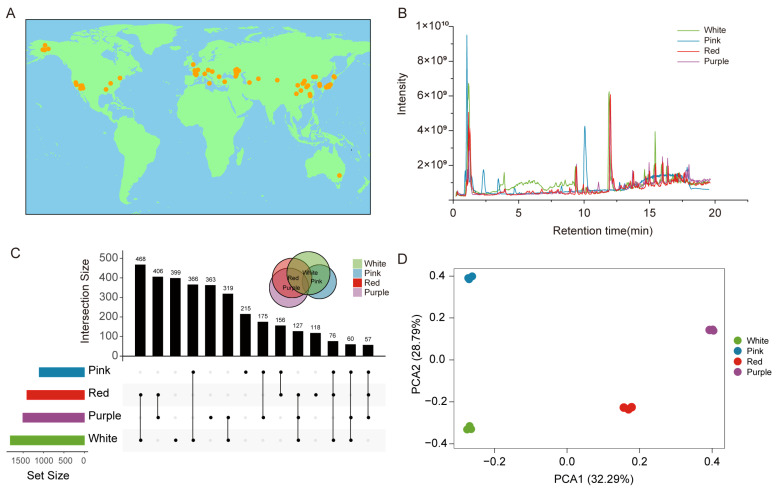
Differential analysis of the metabolome in grapes of different varieties. (**A**) Geographical distribution of 66 grape varieties. (**B**) Total ion chromatography of metabolites in four color grape populations. (**C**) Advanced Venn diagram (UpSet) results for the metabolome data from four color grape populations. (**D**) Principal component analysis (PCA) of total ion chromatography results for four color grape populations.

To comprehensively depict the grape metabolome, we initiated a global germplasm collection effort, gathering 66 valuable grape germplasm resources from renowned grape-producing regions worldwide, including France, Russia, Spain, the United States, Japan, and South Korea (Figure 1A, Supplementary Table S1). The collected grape germplasm encompasses commercial cultivars as well as local varieties and wild species. This diversity ensures that our research can cover a wide range of genetic backgrounds, enabling a better understanding of the metabolic characteristics of different grape varieties. Subsequently, we grouped the berry samples from 66 grape varieties collected worldwide into four mixed samples based on different color combinations (including white, pink, red, and purple mixed samples) and conducted nontargeted metabolomics analysis using LC-MS (Figure 1B).

After the extraction and deduplication of mass spectral signals, a total of 4889 metabolic signals were identified, including 2865 high-resolution mass spectral signals with fragments (Supplementary Table S2). In order to better elucidate the accumulation patterns of metabolites in grape samples of different colors, we utilized metabolite data to generate an advanced UpSet plot, which illustrates the intersection of metabolites among various colored grape varieties (Supplementary Table S3). Through analysis of the differences in metabolic signals between grape samples of different colors, we identified 399, 363, 215, and 188 unique metabolic signals present in white, purple, pink, and red grape samples, respectively (Figure 1C). The results indicate that nontargeted metabolomics analysis of grapes of different colors can aid in the discovery of potential new metabolites that may not be present in individual-colored grapes, and also confirm that this strategy can make a significant contribution to the comprehensive establishment of metabolic databases. Furthermore, statistical analysis was conducted on shared metabolites between samples, revealing a substantial overlap in metabolites between red grape, white grape, and purple grape samples. These findings suggest that shared metabolites among various colored grape varieties may display similar patterns of metabolite accumulation.

Finally, we conducted principal component analysis (PCA) on all samples using these metabolic signals. The PCA results indicated that principal component 1 and principal component 2 accounted for 32.29% and 28.79% of the variance, respectively (Figure 1D), effectively segregating all grape samples by color. Upon closer examination in the PCA plot, it was evident that samples of different colors were separated, with grape samples of distinct colors clearly clustering into four separate groups, suggesting substantial differences in the metabolic profiles among these various grape cultivars.

### 3.2. Identification of Metabolic Signals

In order to further investigate the nutritional and active components in grapes, we proceeded to annotate the metabolic signals present in the samples. To facilitate the identification/annotation of the metabolites detected above, the precursor ions were subjected to a targeted MS2 mode analysis, and fragmentation patterns of them were obtained. Initially, a MS2T library was established using high-resolution data, followed by annotation based on accurate *m*/*z* values, retention times (RTs), and fragmentation patterns. Following a method pioneered by Morreel et al. in plants [27], these features were then utilized for comparison with metabolites found in the literature and databases. Furthermore, unknown metabolic signals were annotated based on existing standards.

**Figure 2:**
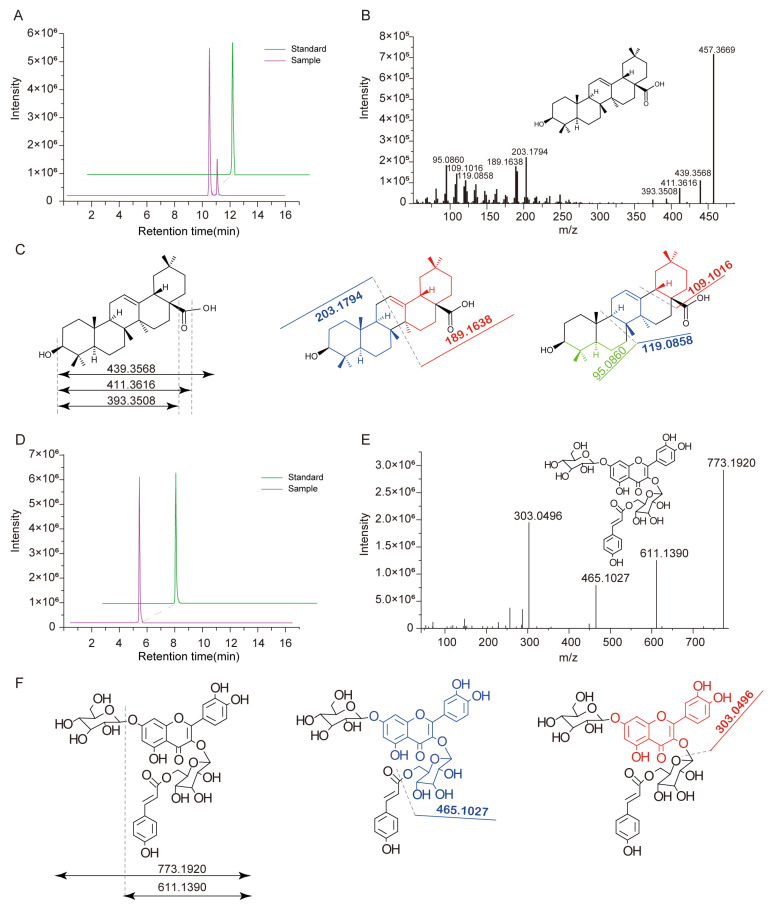
Detection and identification of specific metabolite signs by Q Exactive Focus Orbitrap LC-MS/MS. (**A**) The EIC (extraction) ion chromatogram of *m*/*z* 457.3669 and oleanolic acid authentic standard were detected at 10.737 min. (**B**) MS/MS spectra of *m*/*z* 457.3669 detected at 10.737 min, and its molecular structure. (**C**) Structure and fragmentation pathways of oleanolic acid. (**D**) The EIC (extraction) ion chromatogram of *m*/*z* 773.1920 and quercetin 3-O-beta-(6″-O-E-p-coumaroylglucoside) -7-O-beta-glucoside standard were detected at 5.446 min. (**E**) The mass spectrometry information of *m*/*z* 773.1920 obtained by the targeted MS2 mode, and characterized as quercetin 3-O-beta- (6″-O-E-p-coumaroylglucoside) -7-O-beta-glucoside by comparison of the standard. (**F**) The molecular structure of quercetin 3-O-beta- (6″-O-E-p-coumaroylglucoside) -7-O-beta-glucoside and its general fragmentation rules.

The carbon skeleton of pentacyclic triterpenoids exhibits a variety of modification types, with complex and diverse structures, which have attracted widespread attention due to their multifunctionality. Based on their aglycones, pentacyclic triterpenoids can be mainly divided into four categories including oleanane, ursane, lupane, and friedelane. Among the annotated pentacyclic triterpenoids, oleanane is the most representative [28,29]. The main ion generated by oleanane-type triterpenoids in ESI-MS positive ion mode is *m*/*z* 457.4, which is inferred to be the [M+H-glycosyl-H_2_O]^+^, followed by loss of a carboxyl to form a fragment at *m*/*z* 393.4. This serves as an important characteristic for distinguishing and screening oleanane-type pentacyclic triterpenoids. For instance, PTP0433 was detected with a metabolite signal at Q1 of 457.3669 *m*/*z* at a retention time of 10.737 min (Figure 2A,B). The secondary mass spectra data of PTP0433 show a characteristic peak at *m*/*z* 393.3508, as well as peaks at *m*/*z* 203.1794 and *m*/*z* 189.1638, indicating the presence of the pentacyclic triterpenoid skeleton. Additionally, there are mass spectra signals at *m*/*z* 119.0858, 109.1016, and 95.0860, corresponding to fragment information from isoprene. Considering that the fragmentation pattern resembles that of pentacyclic triterpenoids, it can be inferred that PTP0433 is also a member of this compound class. By comparison with standards, we ultimately annotated PTP0433 as oleanolic acid.

Flavonoids are a class of polyphenolic secondary metabolites found in plants. In practice, the presence of flavonoids in plants often occurs as derivative products, such as glycosylation, methylation, acetylation, and lactonization [30,31]. Among these, glycosylated flavonoids are the most representative. The fragmentation pattern of glycosylated flavonoids involves the potential cleavage of the glycosidic bond (such as O-glycosidic or C-glycosidic) within the molecule, resulting in the separation of sugar molecules from the aglycone to form larger fragments denoted as Y0^+^. One sugar group resulted in a fragment ion [M + H − 162]^+^; this pattern continues for multiple sugar groups and forms characteristic fragment ions. Subsequently, CH3 (15 Da), OCH3 (31 Da), CO (28 Da), and H2O (18 Da) are lost to form different fragments. For instance, a metabolic signal with Q1 at *m*/*z* 773.1920 was detected at a retention time of 5.446 min for PTP0043 in (Figure 2D). The secondary mass spectra of PTP0043 revealed characteristic peaks including the Y0^+^ ion at *m*/*z* 303.0496, as well as peaks at *m*/*z* 465.1027 and 611.1390 (Figure 2E). Considering the difference of 162 Da between the characteristic peaks at *m*/*z* 303.0496 and *m*/*z* 465.1027, and also the difference of 162 Da between the peak at *m*/*z* 611.1390 and Q1 at *m*/*z* 773.1920, we deduce that the substance contains two hexose sugars based on this mass spectrometry analysis. Upon further analysis of the secondary mass spectrometry data, we observed that the feature peak at *m*/*z* 303.0496 corresponded to the mass spectrum information of polyhydroxylated flavonoids. Considering the typical elution times of flavonoids, we inferred that this substance is a glycosylated derivative of flavonoids. Additionally, the characteristic peak at *m*/*z* 611.1390 was 146 Da greater than the peak at *m*/*z* 465.1027, which was consistent with the ionization pattern of the coumaroyl group. Therefore, we hypothesize that the substance is quercetin that has been glycosylated twice and acylated with one coumar. Finally, the substance was annotated as quercetin 3-O-beta-(6″-O-E-p-coumaroylglucoside)-7-O-beta-glucoside by combining the consistency of flavonoid structures, standard comparisons, mass spectrometry fragment information, and retention times (Figure 2F).

In a similar manner, we summarized and annotated the fragmentation patterns of other classes of metabolites. Based on these annotations, we validated our findings using standards and employed the same analytical procedures for sample analysis. By comparing *m*/*z* values, retention times (RTs), and fragmentation patterns with those of standards, we ultimately identified 964 metabolites. These include amino acids and their derivatives, acyl sugars, flavonoids, polyphenols, etc. (Supplementary Table S3). Subsequently, we utilized MRM mode for high-throughput quantitative detection of these identified or annotated metabolites to achieve targeted metabolic metabolite profiling.

### 3.3. Grape Metabolome Profiling and Population Analysis

**Figure 3:**
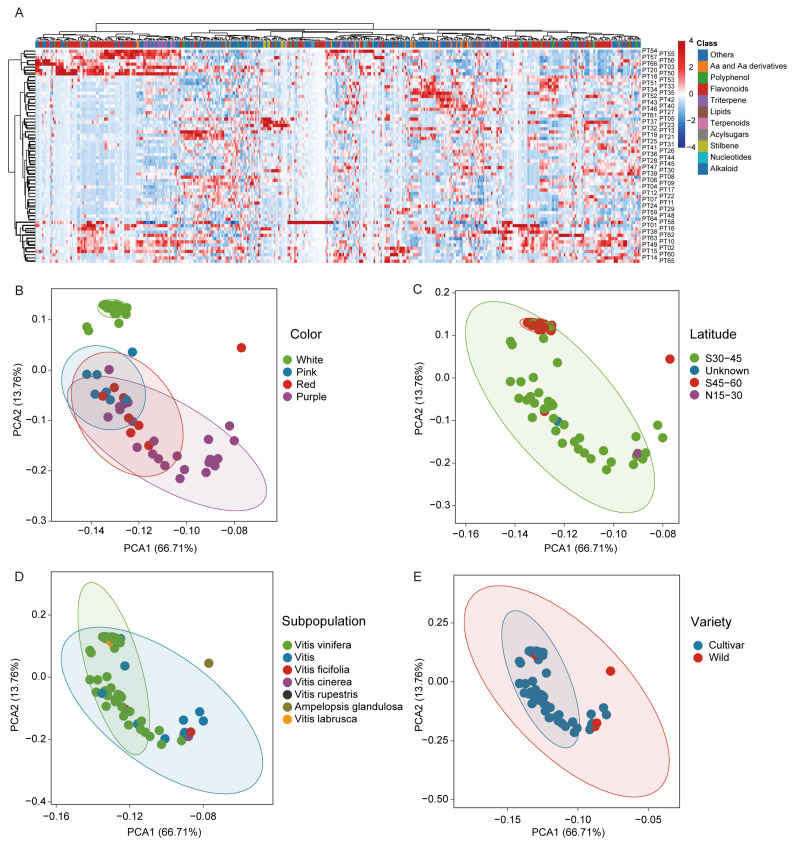
Grape metabolic profiling analysis. (**A**) Hierarchical clustering of 964 metabolites from 66 grape varieties. (**B**–**E**) PCA result for the metabolome data from 66 grape sample.

In order to study the differences in metabolite levels between different grape varieties, we conducted a quantitative analysis of the grape population based on the obtained metabolite database and initiated a preliminary metabolic profile analysis. We first performed hierarchical clustering analysis on 66 grape varieties based on the content of 964 known metabolites. The results show that compared with wild grape varieties, flavonoids, triterpenes, and stilbenes are more abundant in cultivated varieties, suggesting that these compounds may have been selected and accumulated during the process of grape domestication and improvement. It is noteworthy that the flavonoid content in cultivated cultivar species (*Vitis vinifera*) is significantly higher than that of other subspecies, and includes widely reported bioactive active compounds such as kaempferol-3- O -(6-malonyl-glucoside), anacheiloside, and isorhoamnetin3-(6″-p-coumarylglucoside) (Figure 3A). These compounds may have contributed to the exceptional quality of French wines. They not only impart unique flavor and color to the wine but may also have a positive impact on consumer health. The results of previous PCA showed differences in metabolites between grape berries of different colors, which were further confirmed by significant differences observed in hierarchical clustering analysis. For instance, in white, pink, and red grape varieties, the predominant metabolites in these berries are lipids, alkaloids, nucleotides, acyl sugars, stilbenes, and vitamins.

In contrast, the bioactive compounds in purple grapes are more diverse and abundant, including high levels of flavonoids, amino acids and their derivatives, triterpenes, and polyphenols. Among them, flavonoids have excellent antioxidant properties, which can resist the damage caused by free radicals to the human body and reduce the risk of cardiovascular diseases. Meanwhile, triterpenes have anti-inflammatory, antitumor, and other pharmacological activities, while polyphenols have antioxidant, anti-inflammatory, and antitumor biological activities. These diverse metabolites make purple grapes have higher nutritional value and health benefits. Hierarchical cluster analysis reveals significant quantitative differences in metabolites among different grape varieties, indicating that there are significant differences in the types and quantities of metabolites at the species level.

Furthermore, we will conduct principal component analysis (PCA) on the quantitative results of 964 metabolites detected in 66 grape samples. Previous studies have suggested that different regions may impact plant metabolites [32]. The PCA results indicate that the four distinct latitudes cannot differentiate between the 66 grape varieties (Figure 3B). Subsequently, we attempted to classify the samples based on subpopulations and cultivar types; however, the separation effect was not clearly evident on the PCA plot (Figure 3D,E). It is noteworthy that when comparing different colored grape varieties, the PCA results reveal significant differences in metabolites among them. The PCA demonstrates that the first principal component (PC1) and second principal component (PC2) account for 66.71% and 13.76% of total variance, respectively. The clustering of pink, red, and purple grapes in the PCA plot suggests similar metabolic profiles among these color grape varieties. The successful differentiation of white grape varieties from others indicates substantial differences in metabolites between various color types of grapes—possibly due to specific metabolites determining grape berry color.

### 3.4. Metabolic Accumulation Patterns Across Grape Cultivars

#### 3.4.1. Metabolic Diversity of Flavonoids in Grape Varieties

Epidemiological evidence consistently indicates that higher intake of flavonoids is associated with a lower incidence of cardiovascular diseases [33] and reduced cancer risk [34,35]. Polyphenolic compounds in Scutellaria, such as baicalin, baicalein, and wogonin, have been reported to possess anti-inflammatory and anticancer properties. These compounds exert their effects by inhibiting the activity of inflammation-related enzymes, scavenging free radicals, and regulating cell signaling pathways, thereby impacting the growth and apoptosis of tumor cells [36]. Additionally, multi-modified flavonoids have been found to potentially lower blood sugar levels and may have therapeutic effects for diabetes. For instance, the unique cyanidin found in the Centaurea plant can activate the downstream pathway of insulin receptors, lower fasting blood glucose levels, and improve insulin resistance. It is speculated that it plays a role in preventing diabetes by affecting insulin signaling, inhibiting fibrosis, and enhancing antioxidation [37]. Hence, we performed hierarchical clustering analysis on the flavonoids present in 66 distinct grape germplasm berries. Through visualized hierarchical clustering analysis (HCA), we observed significant species and content differences in different colored grape varieties. The hierarchical clustering analysis revealed that metabolites were divided into four clusters. The first cluster had high contents of white, pink, and red grapes with lower accumulation in purple grapes, while the second cluster showed high contents in purple grapes and lower contents in other colors. The third cluster exhibited higher accumulation in purple grapes compared to red and pink grapes but almost none in white grapes, whereas the last cluster had metabolites only present in purple grapes with almost no accumulation in other colored ones. Hierarchical clustering analysis revealed that the first cluster primarily consisted of simple flavonoids, while the second to fourth clusters comprised monosaccharide flavonoids, disaccharide flavonoids, and disaccharide derivatives, respectively. These 66 accessions were divided into three groups; purple grapes were separated from other colored grape varieties. In the first cluster, simple flavonoid metabolite content was comparatively lower than that of other colored grape varieties for purple grapes. As the degree of flavonoid modification increases, the accumulation of flavonoids in purple grapes steadily increases while gradually reducing in other colors, until almost no disaccharide glycosylation-derivative flavonoids could be found in other color grape samples.

**Figure 4:**
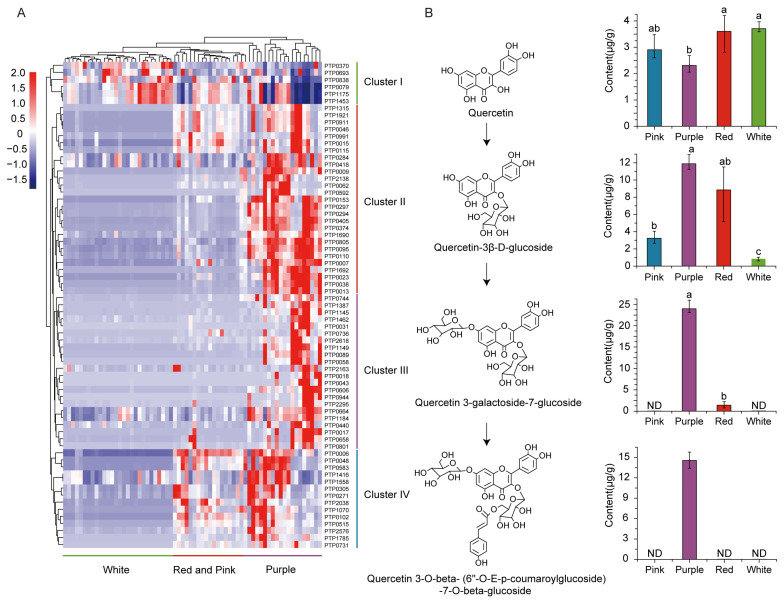
Distribution of flavonoids in different color grape varieties. (**A**) Heat map visualization of the relative difference in flavonoids among different colored grape species. (**B**) The molecular structure and relative content of quercetin and its derivatives in various grape varieties of different colors. Means with different letters (a, b, c) are significantly different at the level of *p* < 0.05. ND: Not Detected.

In addition, in order to further elucidate the accumulation patterns of flavonoids in different grape varieties, we conducted a quantitative analysis of the flavonoid content in four different colored grape varieties. The results showed that differently modified flavonoids exhibited distinct accumulation patterns in these grape varieties (Figure 4B). For instance, quercetin content was slightly lower in purple grapes compared to the other groups, but quercetin-3β-d-glucoside had higher levels in purple grapes than the other three colors. As the degree of modification increased, the content of modified products such as quercetin 3-galactoside-7-glucoside and quercetin 3-O-beta- (6″-O-E-p-coumaroylglucoside)-7-O-beta-glucoside in purple grapes significantly increased compared to the other three groups. Furthermore, they exhibited a higher level of flavonoid-modified products compared to the quercetin. This finding is consistent with previous research, indicating that purple grapes possess stronger antioxidant properties and higher levels of modified flavonoids [38]. These studies collectively suggest that different colored grape varieties demonstrate distinct patterns of accumulation for structurally modified flavonoids.

Overall, our research has revealed significant differences in flavonoids among grapes of different colors. Furthermore, our analysis of modified flavonoids has shown that the levels of modified flavonoids in purple grapes are significantly higher than those in other colored varieties. This indicates distinct patterns of flavonoid accumulation, which may be associated with the potent biological activity functions observed in purple grapes.

#### 3.4.2. Metabolic Diversity of Other Active Compounds in Grape Varieties

**Figure 5:**
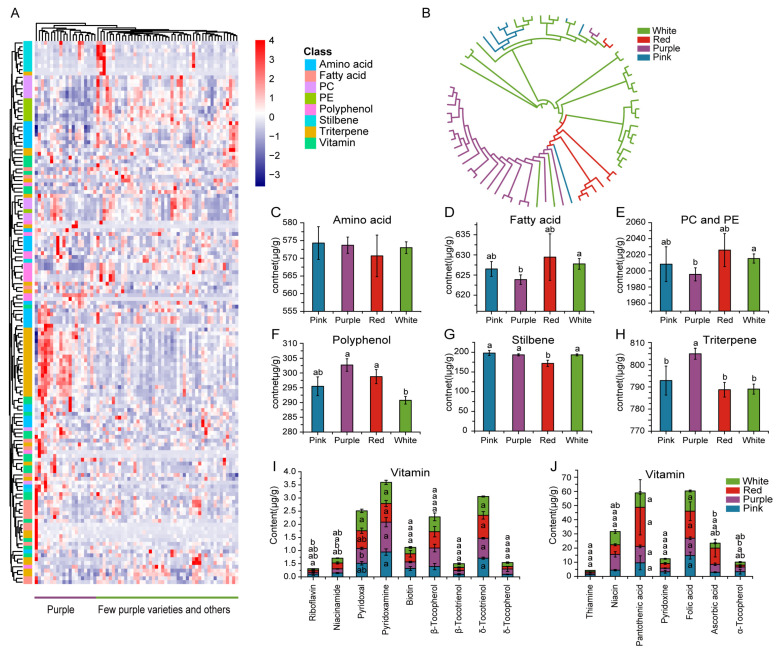
Distribution of other active compounds in different colored species. (**A**) Heat map visualization of the relative difference of amino acid, fatty acids, PC, PE, polyphenol, stilbene, triterpene and vitamins in different colored grape species. (**B**) Neighbor-joining tree of 66 Grape accessions with other active selected metabolites. The tree identifies four subgroups (purple variety, red grape, pink grape and white grape) in different colors. The scale bar indicates the simple matching distance. (**C**–**J**) Contents of metabolites in four color grape varieties. Means with different letters (a, b) are significantly different at the level of *p* < 0.05.

In order to conduct a more comprehensive metabolic profiling analysis of the global grape metabolome, we further explored other potential bioactive compounds present. These bioactive compounds include, but are not limited to, amino acids, vitamins, polyphenols, and other metabolites. Utilizing these metabolites, a phylogenetic tree analysis was performed which revealed that in addition to flavonoids, these metabolites could effectively discriminate between grapes of different colors (Figure 5B). In the course of our research, we conducted a more in-depth examination of the contrast in the content of active compounds between grapes of different colors. In order to provide a more intuitive demonstration of this difference, we opted to perform hierarchical clustering analysis on the aforementioned metabolites (Figure 5A). Through detailed comparison and analysis of the metabolic composition of 66 grape varieties, we categorized them into two primary clusters. Our clustering analysis revealed significant differences in metabolite accumulation between most purple grapes and those of other colors. Specifically, purple grape varieties exhibited higher accumulation of triterpenes and polyphenolic compounds compared to other colored grape varieties. These compounds have important implications for the health benefits and other aspects related to grapes. For instance, triterpenoids exhibit biological activities such as antioxidant and anti-inflammatory effects, while polyphenols are known for their beneficial effects in combating tumors and cardiovascular diseases. Additionally, grapes of other colors are rich in fatty acids, PC, and PE, as well as small amounts of amino acids. These components play a significant role in human metabolism and growth development.

In addition, in order to further explore the accumulation patterns of these other active compounds, we conducted a relative quantification of the active compounds in four mixed samples of different colors. We specifically examined the distribution of each type of substance in grapes of different colors, such as the notably lower content of FAs in purple grapes compared to the other three grape varieties (Figure 5A). Additionally, we observed lower levels of fatty acids and phospholipids, which also suggest that moderate consumption of red wine does not impact body fat content, consistent with the results from the “French paradox”. Through comparative analysis of the accumulation patterns of these metabolites, different accumulation patterns from those of flavonoids were observed. Specifically, amino acid abundance was slightly lower in mixed samples containing purple grapes compared to those with pink grapes. However, there were similarities in the accumulation process for stilbenes across all color grape mixed samples with little variation in their contents. It is noteworthy that stilbenes include resveratrol, which has been associated with the “French paradox”. This further indicates that differences in biological activity functions among different grape varieties may not be solely attributed to resveratrol. Therefore, the bioactivity of resveratrol remains controversial and requires further in-depth research. Additionally, in the relative quantification results, we found that the purple grape sample had the highest content of polyphenols and triterpenes with antioxidant activity, significantly higher than other color samples. This result further confirms the advantage of purple grapes in antioxidant active compounds and provides a valuable clue for subsequent research. Finally, considering the importance of vitamins in the nutritional composition and flavor profile of grapes, we conducted absolute quantitative analysis of various vitamins in the four colored grape samples (Supplementary Table S4). The study results revealed significant variations in the vitamin levels among grapes of different colors. Specifically, the red grapes exhibited notably higher levels of folic acid (19.10 μg/g), L-ascorbic acid (11.47 μg/g), and nicotinamide (11.16 μg/g) compared to other colored samples. The pink grapes are relatively rich in riboflavin, with a content of (0.11 μg/g), which is beneficial for eye protection. On the other hand, purple grapes exhibit superior anti-inflammatory and antioxidant properties, particularly in the accumulation of niacin, pyridoxamine, α-tocopherol, and β-tocopherol at levels of 11.16 μg/g, 1.14 μg/g, 0.71 μg/g, and 0.20 μg/g, respectively. Based on our research and analysis, we conclude that purple grapes possess significant natural advantages in terms of antidisease activity. Compared to grapes of other colors, purple grapes contain higher levels of antioxidants and biologically active compounds. In addition to complexly modified flavonoids, other compounds also contribute to the stronger biological activity of purple grapes such as polyphenols, triterpenes, and pyridoxamines. These components endow purple grapes with enhanced capabilities for disease prevention and resistance while providing more pronounced health benefits and wellness functions for individuals.

In conclusion, grapes of different colors exhibit significant differences in terms of their biological activity. A thorough investigation into these variances can enhance our understanding of the nutritional value of grapes and enable us to offer consumers a wider variety of healthy and delicious grape cultivars. Furthermore, these research findings can serve as valuable references for grape growers, assisting them in determining cultivation strategies aimed at enhancing the quality of grapes.

## 4. Discussion

Grapes are an indispensable and significant component of the human diet, closely associated with health and well-being. They are nutritionally equivalent to vegetables, providing essential polyphenols, amino acids, and vitamins necessary for the human body [39]. Resveratrol, a stilbene compound, has been extensively studied in various diseases and may play a potentially important role. Research on this compound originated from the “French paradox”, which described the lower incidence of cardiovascular diseases among French people despite their high-fat diet compared to other regions with low-fat diets [17]. However, recent studies suggest that resveratrol may not be beneficial in treating cardiovascular and cerebrovascular diseases as well as cancer. Therefore, it is imperative to conduct comprehensive metabolic profiling of grapes to identify bioactive compounds truly relevant to human health.

In this study, we utilized nontargeted LC-MS technology to detect 4889 metabolic signals in 66 grape samples. By comparing the hierarchical clustering results of different colored grape samples, we observed significant differences in metabolic characteristics between various grape varieties Figure 1D and Figure 2B). To further investigate the metabolic variances among different colored grapes, a targeted metabolomics approach revealed that purple grapes exhibited a richer diversity of metabolites, including numerous beneficial health compounds. Furthermore, the UpSet plot demonstrated a substantial number of unique metabolic signals in each color of grape sampled (Figure 1C). To the best of our knowledge, a comprehensive investigation into the metabolic profiles of different colored grape varieties has not yet been undertaken. Utilizing principal component analysis (PCA), 66 grape varieties were differentiated based on color, revealing a high degree of consistency in metabolites within grapes of the same color and distinct contrasts between those of different colors. These differences were found to be independent of geographical regions, subspecies, or cultivation methods. Based on these findings, we hypothesize that the variations in metabolic composition among different colored grape varieties may be linked to specific physiological and biochemical changes experienced during their maturation process. These changes are likely influenced by both genetic and environmental factors, resulting in differentiation in metabolite composition among different colored grape varieties. Color as a distinguishing feature for metabolomic differentiation may more directly reflect the physiological status and metabolic activities of grape berries during maturation.

Despite significant technological advancements in nontargeted metabolomics using LC-MS, which can elucidate metabolic events occurring in plants by detecting secondary metabolites, one of the most challenging technical issues encountered in LC-MS metabolomics is the annotation of numerous unknown metabolites. We previously established a novel and comprehensive targeted metabolomics approach for high-throughput quantification of hundreds of targeted metabolites, which has been widely utilized in rice research and enables the characterization of new metabolites [40,41]. Using this method, we identified many bioactive metabolites in this study, including various modified forms of flavonoids and triterpenoids (Figure 2A).

In our in-depth investigation of the metabolic variances among grapes of different colors, we employed a targeted metabolomics approach to achieve more precise and thorough results. Our study revealed that purple grapes exhibit a rich diversity of metabolites with distinct differences compared to other colored grapes, showing higher levels of health-promoting compounds (Figure 3A). These beneficial metabolites include flavonoids, amino acids and their derivatives, triterpenes, and polyphenols. They are notably abundant in purple grapes and have been widely acknowledged for their biological activities by scientific research. This research outcome not only enhances our understanding of the metabolic disparities between grape varieties of different colors but also provides crucial insights for further exploration into the nutritional value and sensory attributes of grape berries.

Flavonoids are one of the main secondary metabolite types and has significant relevance for grape quality. A recent study unveiled a significant correlation between the composition and quantity of flavonoids, alongside the antioxidant potential, across 24 European grape (*V. vinifera*) cultivars [41]. However, little is known about the differences in flavonoid composition between different colored grape populations. Our metabolome analysis revealed that purple grapes contain higher levels of multiple modified flavonoids than other colored grapes (Figure 4A), which is in line with what is expected of the antioxidant capacity of red wine [42,43]. These various modified flavonoids not only enhance the grape’s natural antioxidant defense mechanisms but may also be a contribution to the health care function and flavor complexity of wine. In addition, the presence of these compounds may be related to the health benefits of red wine in the prevention of cardiovascular disease, the so-called “French paradox” photograph echo.

In addition to flavonoids, vitamins, amino acids, fatty acids, and polyphenols are also crucial components of the biological activity of grape fruit [44]. However, there has been limited research on the metabolic profile differences between grape fruits of different colors. Through comparative metabolomics analysis, it was observed that purple grape varieties exhibited higher levels of most triterpenes and polyphenols compared to the other three colored grapes. Conversely, the other three colored grape varieties contained higher levels of fatty acids, PC, and PE. Notably, most wine grapes are purple and contain high levels of health-promoting active compounds such as triterpenes, polyphenols, and flavonoids beneficial for human health. Furthermore, in comparing vitamin content among various colored grape varieties, it was found that purple grapes demonstrated outstanding anti-inflammatory and antioxidant properties with notably higher accumulation of niacin, pantothenic acid, pyridoxamine, and tocopherol. These compounds effectively prevent disease while also preventing the oxidation of the grape fruit. These results indicate that, in addition to various flavonoids, other compounds confer strong biological activity to purple grapes, playing a significant role in their growth and development as well as the quality formation and health benefits for humans. Additionally, our work reveals differences in nutrient accumulation patterns among different types of grape varieties, suggesting potential complementarity between nutrients in differently colored grapes, providing valuable insights into their metabolic characteristics. Research on natural variation in metabolomes has made significant progress for major crops such as rice and maize, offering genetic biochemical insights applicable to analyzing natural variation within the metabolome of grapes [45]. Our study of natural variation in grape metabolites revealed that triterpenes were significantly clustered into distinct groups, and similar accumulation patterns were observed between these clusters and other metabolite groups (Figure 5A), a finding supported by the results of the neighbor tree analysis. In summary, this discovery will greatly benefit future grape breeding efforts.

However, our study also has some limitations. Although we identified a large number of metabolites that may have biological activity, the precise biological functions and their impact on human health still require further validation through animal models and clinical studies. Additionally, our study primarily focused on the metabolite composition of grape berries, while the metabolic characteristics of other tissues such as grape leaves and stems have not been thoroughly investigated.

Our research findings have important implications for grape breeding and consumption. Firstly, by understanding the metabolic characteristics of different colored grape varieties, breeders can selectively choose and cultivate new varieties rich in beneficial active compounds. Secondly, consumers can make rational choices and combinations of different colored grape varieties based on their personal health needs and taste preferences to obtain more comprehensive nutrition and health benefits. In summary, our study has revealed metabolic differences among different colored grape varieties using metabolomics methods, providing new insights for grape breeding, consumption, and product development. Future research should further explore the biological functions of these metabolites and their impact on human health to maximize the efficient utilization of grape resources and their health value.

## 5. Conclusions

In this study, we demonstrate that using the combination of a nontargeted and targeted metabolomics approach, we can assess the comprehensive metabolic profile and natural variation in metabolites in grapes. The combination of nontargeted metabolic profiling and PCA was practicable in classifying colored grape species. The analysis revealed that flavonoids, polyphenols, and triterpenes were predominantly present in the purple grape variety, whereas amino acids and PC were primarily concentrated in the other three colored grapes. Vitamins were also determined in four different color grape populations, and the content of vitamins attained the highest levels in pink grape populations.

## Data Availability

The original contributions presented in the study are included in the article/Supplementary Material, further inquiries can be directed to the corresponding authors.

## Supplementary Materials

The following supporting information can be downloaded at: https://www.mdpi.com/article/10.3390/foods13233716/s1, Table S1: List of 66 grape accessions used in this study; Table S2: Information of 4889 metabolic signals detected in grape fruit mix samples using non-targeted metabolomics detection by LC-MS/MS with Fullscan+ddMS2; Table S3: The identified metabolites in this study; Table S4: Absolute quantitative vitamins (μg/g).

## Abbreviations

LC-MS	liquid chromatography–tandem mass spectrometry
RES	resveratrol
PMFs	potent anticancer compounds
MbA	antidiabetes flavonoid glycosides
DAMs	differentially accumulated metabolites
RT	retention times
PCA	principal component analysis
PC	phosphatidylcholine
PE	phosphatidylethanolamine
FA	fatty acid
